# Research on Fault Prediction Method of Elevator Door System Based on Transfer Learning

**DOI:** 10.3390/s24072135

**Published:** 2024-03-27

**Authors:** Jun Pan, Changxu Shao, Yuefang Dai, Yimin Wei, Wenhua Chen, Zheng Lin

**Affiliations:** 1 Zhejiang Province’s Key Laboratory of Reliability Technology for Mechanical and Electronic Product, Zhejiang Sci-Tech University, Hangzhou 310018, China; 2 Hangzhou Xizi Iparking Co., Ltd., Hangzhou 311103, China

**Keywords:** fault prediction, elevator door, GNN, LSTM, transfer learning

## Abstract

The elevator door system plays a crucial role in ensuring elevator safety. Fault prediction is an invaluable tool for accident prevention. By analyzing the sound signals generated during operation, such as component wear and tear, the fault of the system can be accurately determined. This study proposes a GNN-LSTM-BDANN deep learning model to account for variations in elevator operating environments and sound signal acquisition methods. The proposed model utilizes the historical sound data from other elevators to predict the remaining useful life (RUL) of the target elevator door system. Firstly, the opening and closing sounds of other elevators is collected, followed by the extraction of relevant sound signal characteristics including A-weighted sound pressure level, loudness, sharpness, and roughness. These features are then transformed into graph data with geometric structure representation. Subsequently, the Graph Neural Networks (GNN) and long short-term memory networks (LSTM) are employed to extract deeper features from the data. Finally, transfer learning based on the improved Bhattacharyya Distance domain adversarial neural network (BDANN) is utilized to transfer knowledge learned from historical sound data of other elevators to predict RUL for the target elevator door system effectively. Experimental results demonstrate that the proposed method can successfully predict potential failure timeframes for different elevator door systems.

## 1. Introduction

An elevator is a kind of special equipment widely used in all kinds of public places, and its running condition is directly related to the safety of passengers. The elevator door system is a pivotal component within this infrastructure, with its functionality affecting not only the passenger experience but also potentially leading to severe accidents. For instance, an abnormality in the opening mechanism can result in passengers being trapped, while failure to close before elevator movement may precipitate shearing or pinching incidents. According to statistical data, more than 80% of all elevator accidents are attributed to door system malfunctions [1]. To determine the state of elevator door systems, KIM et al. [2] proposed a knowledge-based feature operation. They utilized a variational autoencoder with restricted latent space and Bayesian optimization to obtain a margin-maximized hyperspace (MMH). This approach enables accurate fault classification for elevator doors. Koutsoupakis et al. [3] constructed a physical system multibody dynamics (MBD) model of elevator door tracks to acquire relevant fault data. They then introduced a novel CM framework based on Convolutional Neural Networks (CNN) for detecting damage in elevator door tracks. CHAE et al. [4] used the control state information for the fault diagnosis of an elevator door, and the state of elevator door control cabinets was used as information to classify their operating states. However, all these studies focusing on diagnostic methods for elevator door systems fail to provide predictive warnings before actual failure. In light of accident prevention, the need for proactive fault prognostics for elevator doors emerges as a critical and unignorable issue.

In the domain of fault prediction research, Long Short-Term Memory (LSTM) models have been extensively applied for tackling time series forecasting challenges. They have evidenced commendable performance in predicting failures within various mechanical components, such as rolling gearboxes [5], turbine engines [6], and rolling bearings [7]. Concurrently, Graph Neural Networks (GNN) have seen gradual adoption in the fields of fault diagnosis [8] and fault prognostics [9] over recent years. GNN was originally introduced by Scarselli et al. [10]. Unlike conventional deep learning architectures, GNN is designed to inherently accommodate the interdependencies among data points. The structural geometry inherent in data is exploited by GNN to extract and provide supplementary feature information that can enhance predictive performance. While these methodologies have demonstrated commendable outcomes within the domain of fault prediction, their effectiveness relies heavily on the availability of ample amounts of data and a relatively homogeneous distribution thereof. However, they do not readily address scenarios characterized by limited datasets or those involving cross-machine applications where significant discrepancies in data distributions occur.

Transfer learning refers to the application of knowledge acquired in one domain (source domain) to adapt to a new task in another domain (target domain), which can solve the problem of the small amount of data and knowledge across machines to a certain extent. Several fault prediction methods based on transfer learning have been proposed. Sun et al. [11] introduced a deep transfer learning method that employs three transfer strategies—weight transfer, hidden feature transfer learning, and weight updating. These strategies enable the model trained on the historical fault data of a tool to be transferred to a new object. Wan et al. [12] employed an improved version of the deep residual network (ResNet) as a feature extractor, utilizing multi-kernel maximum mean discrepancy (MK-MMD) and multiple domain discriminators to align the marginal and conditional distributions between the source and target domains, thereby achieving cross-domain fault diagnosis for bearings. Cheng et al. [13] proposed an intelligent method based on dynamic domain adaptation (DDA), which constructs both a dynamic distribution adaptation network (DDAN) and a dynamic adversarial adaptation network (DAAN) to automatically extract degradation features invariant to different operating conditions, thus addressing the issue of inconsistent distributions caused by varying working conditions and enabling predictions of RUL for machinery under multiple operating conditions. He et al. [14] introduced a novel cross-domain predictive maintenance approach that utilizes a joint MMD metric to measure the differences in probability distributions between the two domains and incorporates manifold regularization to ensure the continuity and comparability of the extracted degradation features. This method enables RUL prediction for the target domain data using failure data from the source domain. Zhu et al. [15] proposed a Bayesian semi-supervised transfer learning framework based on active querying for intelligent fault prediction. This approach utilizes transfer learning to achieve RUL prediction across entirely different machines with limited data. Mao et al. [16] presented a novel selective transfer learning method for cross-machine RUL prediction, particularly when there are significant differences in data distribution and fault evolution characteristics.

In addition, among the various diagnostic and predictive techniques, vibration and current-based monitoring have been in use for an extended period and are considered the most mature methods. However, these approaches typically necessitate physical contact between sensors and the monitored equipment, which can pose constraints in specific operational environments. In recent years, sound-based monitoring has garnered substantial interest from researchers [17,18,19]. This is because the sounds emitted by equipment in different health states vary, serving as a leading indicator for fault identification.

This paper primarily investigates a fault prediction method for elevator door systems that incorporates transfer learning. Firstly, the sound-based GNN-LSTM fault prediction method is proposed by using deep learning knowledge. Then, the GNN-LSTM-BDANN model is constructed by combining the transfer learning method which uses the DANN based on Bhattacharyya distance improvement (BDANN), and the RUL prediction of the target elevator is realized by using other elevator historical data, which solves the problem that the whole life cycle data of the elevator is difficult to obtain, and provides a favorable guarantee for the fault prediction and preventive maintenance of the elevator door system.

## 2. Fault Characterization and Feature Extraction

### 2.1. Fault Characterization of an Elevator Door System

**Figure 1:**
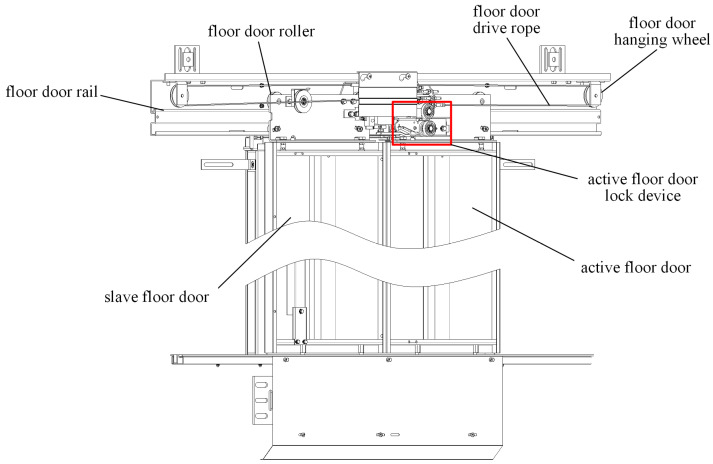
Elevator floor door structure.

**Figure 2:**
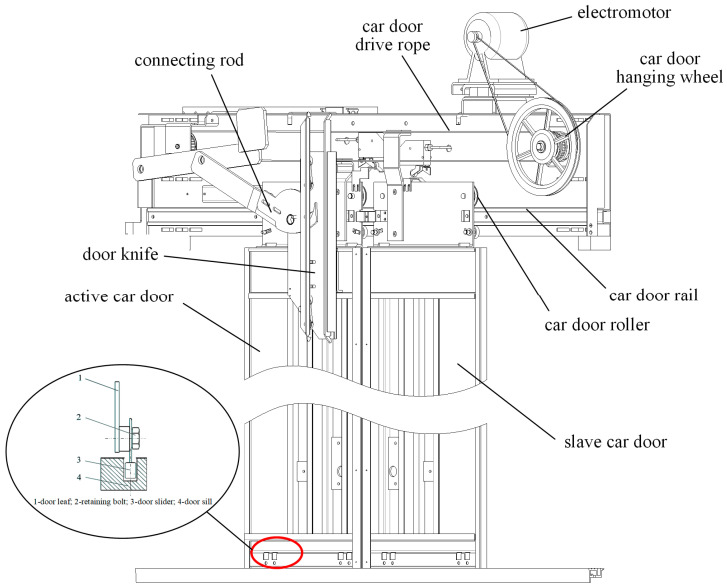
Elevator car door structure.

The structure of the elevator door system is shown in Figure 1 and Figure 2. Mechanical failures are primarily attributed to component wear, loosening, and obstruction by foreign objects, involving issues with components such as guide rails, rollers, door panels, and door locks. These malfunctions represent one of the main reasons for the abnormal operation and safety hazards within the system.

The opening and closing of elevator doors rely on the smooth operation of guide rails and rollers. Faults in the tracks or pulleys may result in sluggish door movements or the generation of abnormal sounds, such as friction noise, scraping sounds, or unusual noises. Friction noise may indicate surface wear on the tracks or rollers, while scraping sounds may suggest the presence of impurities or foreign objects between the tracks and rollers.

The electromotor in the elevator door system drives the movement of the doors, and electromotor issues may lead to abnormal door opening or closing speeds, unstable movements, or complete stoppage. Electromotor malfunctions may produce abnormal sounds, including noise, vibrations, or collisions within the electromotor rotor.

The tracks and sliders at the bottom of elevator doors are crucial components to ensure the accurate trajectory of door movements. Mechanical faults may result in abnormal wear or loosening of these components. Relevant sound signals may include rolling friction noise, collision sound, or vibration sound.

Among the elevator failures, wear-induced malfunctions are a focal point of attention, as they directly relate to the reliability and safety of the elevator door system. Wear occurs due to the continuous friction on the surfaces of components, gradually leading to material degradation and shape changes. On critical components such as guide rails and rollers, wear can significantly impact the smooth movement of the door. Additionally, the bottom track and sliders of the elevator door are susceptible to abnormal wear, causing instability in the motion of the door panels. Therefore, in this paper, we will characterize the wear degradation of components such as guide rails, rollers, bottom track, and sliders through experiments, monitoring, and analysis. We will present the wear degradation trend as the RUL of the elevator door system, providing a basis for the maintenance and improvement of the elevator door system.

Due to the complex internal structure and frequent operation of the elevator door system, it is challenging to find a sensor installation position inside it that neither interferes with the movement of relevant components nor affects the sensor’s data collection. Sound signal collection is a non-intrusive monitoring method, as the installation of sound sensors does not require physical intervention or modification of the elevator door system. This helps avoid any impact on the normal operation of the system, reducing maintenance and monitoring costs. The sound signal also contains a wealth of status information, which is essential for timely detection of potential fault signs, real-time monitoring, and maintenance, helping to reduce the possibility of sudden failure. Furthermore, from the analysis of the main mechanical failures of the elevator door system mentioned above, it is evident that sound signals can provide comprehensive information about the operational status of the system, including data on mechanical components such as friction, vibration, and impacts. This comprehensive information assists maintenance personnel in fully understanding the system’s operational condition, identifying potential faults in advance, and taking preventive maintenance measures, thereby reducing repair time and maintenance costs.

Utilizing sound signals for characterizing mechanical faults in elevator door systems fully leverages their non-intrusiveness, real-time capabilities, and anomaly pattern recognition advantages. This enhances the sensitivity of system monitoring and the efficiency of maintenance, ensuring the reliability and safety of the elevator door system.

### 2.2. Feature Extraction

Sound quality is an evaluation metric proposed based on traditional noise assessment indicators, aiming to provide a more human-centered subjective assessment. Its objective is to quantitatively evaluate subjective feelings, using psychoacoustic models to establish complex relationships between physical quantities (such as sound pressure, sound power, frequency, etc.) and perceptual quantities (such as loudness, sharpness, roughness) for the accurate capture of transient changes in frequency and time domain characteristics caused by the occurrence of faults [20]. The main sound quality parameters are A-weighted sound pressure level (dBA), loudness, sharpness and roughness.

The sound pressure level is calculated as follows [21]:

(1) $$p_{e}=\sqrt{\frac{1}{T}\int_{0}^{T}p^{2}(t)dt}$$

(2) $$L_{p}=20\mathrm{lg}\frac{p_{e}}{p_{0}}$$

where p(t) denotes the instantaneous sound pressure and $p_{0}=2*10^{-5}\;\mathrm{Pa}$ denotes the reference sound pressure. A-weighted sound pressure level results are closer to the perception of the human ear, better reflecting the subjective perception of the sound by the human ear, and therefore are widely used. A-weighted sound pressure level is expressed in units of dB(A).

Based on Zwicker’s theory, calculate the loudness, sharpness, and roughness of acoustic signal [22].

Loudness:

(3) $$n(z)=0.08{\left(\frac{E_{T0}}{E_{0}}\right)}^{0.23}[{\left(0.5+\frac{E}{2E_{T0}}\right)}^{0.23}-1]$$

(4) $$N=\int_{0}^{24Bark}n(z)dz$$

where $E_{T0}$ denotes the sound threshold in a quiet environment, $E_{0}$ denotes the reference excitation value, E denotes the excitation of the calculated sound signal, n(z) is the loudness of each frequency band, and N is the total loudness of the signal, expressed in *sone*.

Sharpness:

(5) $$S=k\frac{\int_{0}^{24Bark}n(z)*z*g(z)dz}{N}$$

(6) $$g(z)=\left\{\begin{array}{cc} 1, & z\leq 16\\ 0.0625*e^{0.1733z}, & z>16 \end{array}\right.$$

where k is the weighting factor, generally taken as 0.11, g(z) denotes the weighting function for different critical frequency bands, s(z) is the sharpness of each frequency band, and S is the total sharpness of the signal, expressed in *acum*.

Roughness:

(7) $$R=0.3f_{\mathrm{mod}}\int_{0}^{24Bark}\Delta L_{E}(z)dz$$

(8) $$\Delta I_{E}(z)=20\log_{10}(\frac{n_{\max}(z)}{n_{\min}(z)})$$

where $f_{\mathrm{mod}}$ is the modulation frequency, $\Delta I_{E}$ denotes the amount of variation in feature loudness, $n_{\max}(z)$ and $n_{\min}(z)$ denote the maximum and minimum values of the feature loudness in each feature frequency band, and R is the total roughness of the signal, expressed in *asper*.

## 3. Model Construction

**Figure 3:**
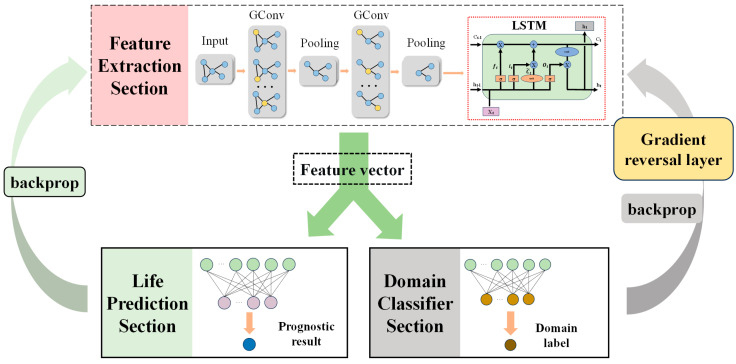
GNN-LSTM-BDANN structure.

Due to the difficulty in obtaining full lifecycle data for each elevator door system, this paper proposes a scheme to predict the remaining useful life of a target elevator door system using historical data from other elevators. To realize this scheme, this paper constructs an improved GNN-LSTM-BDANN fault prediction model based on GNN and LSTM, combined with a Domain-Adversarial Neural Network (DANN) of transfer learning method. The model structure is shown in Figure 3.

### 3.1. GNN-LSTM Feature Extraction Section

The traditional deep learning methods can be effective in capturing hidden features of regular data (e.g., images and time series), but most of them ignore interdependencies between data [9]. GNN, in order to consider the interdependence between data, represents data in the form of graphs in graph theory. In graph data, the relationship between nodes is reflected in the connected edges, and the weights of the edges reflect the strength of this relationship. GNN can propagate the node information through the edges of the graph and learn the useful node or graph information.

**Figure 4:**
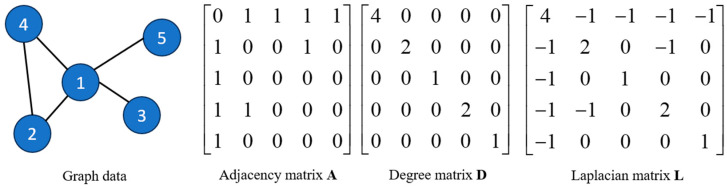
Graph data and three matrices.

In the signal represented by graph, the graph G=G(X,A,E), where $X\in R^{n\times d}$ represents the node identity matrix, E is the set of edges, n is the number of nodes, and d is the feature length. $A\in R^{n\times n}$ denotes the adjacency matrix for the undirected graph, $A_{ij}$ denotes the edge connecting node $v_{i}$ to $v_{j}$ for the directed graph, and $A_{ij}$ denotes the edge from node $v_{i}$ to $v_{j}$. In addition to the adjacency matrix, the graph can also be represented by the Laplacian matrix and the degree matrix, which can be obtained using Equation (9). Figure 4 shows the graph data and the graphical representation of the three matrices above.

(9) $$\left\{\begin{array}{l} L=D-A\\ D_{ii}=\sum_{j}A_{ij} \end{array}\right.$$

**Figure 5:**
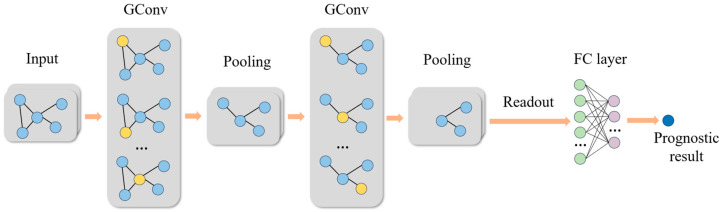
The framework for graph-level fault prognostics task.

When GNN models are employed for predictive tasks, they typically use a graph-level task architecture, where the entire graph is treated as a single sample. The model first learns node representations and then obtains the representation of the entire graph. This architecture consists of GConv layers, graph pooling layers, readout layers, and a fully connected layer, as shown in Figure 5.

LSTM is a special RNN structure that can overcome the problem of long-term dependence and disadvantages of gradient vanishing and explosion during the training process of RNN [23].

**Figure 6:**
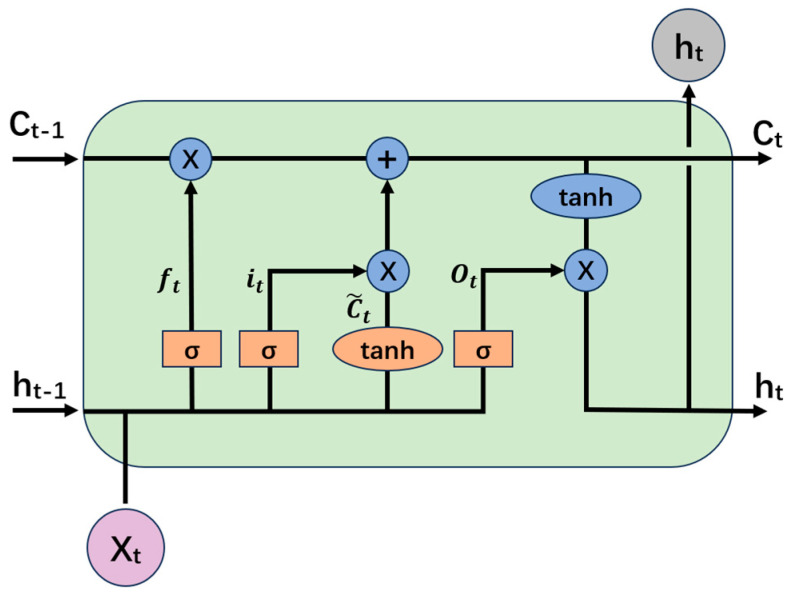
The cell structure of LSTM.

The cell structure of LSTM is shown in Figure 6. The special feature of LSTM is that it decides how much to forget, how much to remember, and how much to output at each point in time through forgetting gate $f_{t}$, input gate $i_{t}$, and output gate $O_{t}$, and finally passes this state all the way through, so that it does not forget distant and important information, and that it does not take nearby unimportant information too seriously.

Given the input $x_{t}$, the output of the last moment $h_{t-1}$, and the state of the last moment $C_{t-1}$, the update process for each time step of the LSTM is as follows:

(10) $$i_{t}=\sigma (W_{ii}x_{t}+b_{ii}+W_{hi}h_{t-1}+b_{hi})$$

(11) $$f_{t}=\sigma (W_{if}x_{t}+b_{if}+W_{hf}h_{t-1}+b_{hf})$$

(12) $${\tilde{C}}_{t}=\tanh(W_{i\tilde{C}}x_{t}+b_{i\tilde{C}}+W_{hg}h_{t-1}+b_{h\tilde{C}})$$

(13) $$O_{t}=\sigma (W_{iO}x_{t}+b_{iO}+W_{hO}h_{t-1}+b_{hO})$$

(14) $$C_{t}=f_{t}⊙C_{t-1}+i_{t}⊙{\tilde{C}}_{t}$$

(15) $$h_{t}=O_{t}⊙\tanh(C_{t})$$

where W is the corresponding weight matrix, b is the corresponding bias vector, and σ is the sigmoid activation function for compressing the input in the range [0, 1].

Utilizing GNN-LSTM as the feature extractor, GNN effectively captures the graph structural relationships among the data and LSTM aids in considering the temporal dependencies of feature data. By combining the strengths of both, the GNN-LSTM can generate more representative and richer feature representations. This enhances the model’s sensitivity to changes in the system state, allowing it to better capture the state of the predictive object at different time points and comprehensively capture the evolution of the data.

Its structure is shown in the feature extraction section in Figure 3. The sound quality feature dataset of the sound signal obtained above is constructed into a graph dataset that can be learned by GNN. After that, abstract features in the dataset are extracted through multiple GConv layers and pooling layers. At last, LSTM is used to find the temporal variation features in these abstract features and output a set of feature vectors.

### 3.2. DANN Improved Based on Bhattacharyya Distance

**Figure 7:**
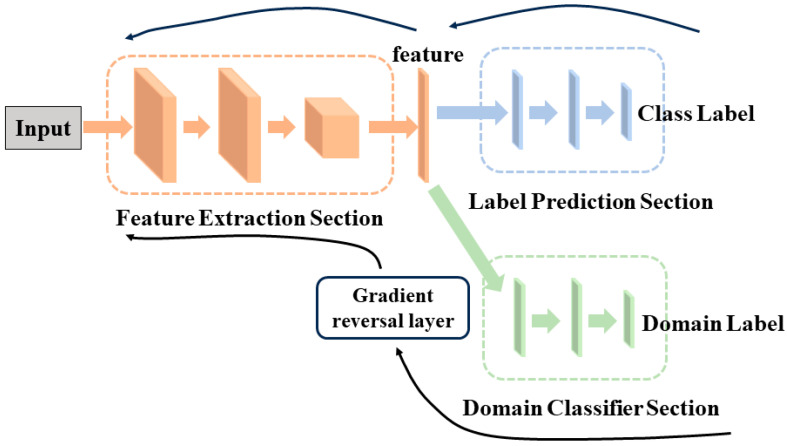
The structure of DANN.

Transfer learning is a machine learning method that utilizes knowledge and features learned from one task to improve learning performance on another related task. In transfer learning, the dataset with existing prior knowledge is referred to as the source domain, while the dataset where the algorithm needs to learn new knowledge is known as the target domain. When there is a difference in data distribution between the source and target domains but the tasks are the same, transfer learning is termed domain adaptation [24]. DANN builds upon domain adaptation by introducing the concept of generative adversarial learning. It employs an iterative adversarial feedback mechanism between a feature extractor and a domain discriminator to reduce the distribution gap between the source and target domains [25]. The structure of DANN is depicted in Figure 7.

From Figure 7, we can observe that DANN is composed of three parts: the feature extraction section $G_{f}(x;\theta_{f})$, the label prediction section $G_{y}(f;\theta_{y})$, and the domain classification section $G_{d}(f;\theta_{d})$. Here, $\theta_{f}$, $\theta_{y}$, and $\theta_{d}$ represent the parameter vectors for each respective section. The input is denoted as x, and f represents the feature vector. During the training of DANN, the class labels and the domain labels of the source domain data are known, while the domain labels of the target domain data are known, but the class labels are unknown. In order to achieve accurate classification on the source domain dataset while also confounding the source domain dataset with the target domain dataset, the loss function of DANN can be defined as follows:

(16) $$\begin{array}{cc} E(\theta_{f},\theta_{y},\theta_{d}) & =\sum_{\begin{array}{l} i=1,\cdots ,N\\ d_{i}=0 \end{array}}L_{y}(G_{y}(G_{f}(x_{i};\theta_{f});\theta_{y}),y_{i})\\ & -\lambda \sum_{i=1,\cdots ,N}L_{d}(G_{d}(G_{f}(x_{i};\theta_{f});\theta_{d}),y_{i})\\ & =\sum_{\begin{array}{l} i=1,\cdots ,N\\ d_{i}=0 \end{array}}L_{y}^{i}(\theta_{f},\theta_{y})-\lambda \sum_{i=1,\cdots ,N}L_{d}^{i}(\theta_{f},\theta_{d}) \end{array}$$

where $L_{y}$ represents the loss value for label prediction, $L_{d}$ represents the loss value for domain label prediction, and λ denotes the weight. The optimal solutions of related parameters are as follows:

(17) $$\left({\hat{\theta }}_{f},{\hat{\theta }}_{y})=\arg\underset{\theta_{f},\theta_{y}}{\min}\;E(\theta_{f},\theta_{y},{\hat{\theta }}_{d}\right)$$

(18) $${\hat{\theta }}_{d}=\arg\underset{\theta_{d}}{\max}\;E({\hat{\theta }}_{f},{\hat{\theta }}_{y},\theta_{d})$$

where argmin denotes the value of the variable that minimizes the expression following it, and argmax denotes the value of the variable that maximizes the expression following it.

To unify the gradient directions of label prediction loss and domain classification loss, the Gradient Reversal Layer (GRL) is introduced in the DANN structure, which automatically reverses the gradient direction during the backpropagation process. The forward and backward propagation can be described using the following equations:

(19) $$R_{\lambda }(x)=x$$

(20) $$\frac{dR_{\lambda }}{dx}=-\lambda I$$

where I is the identity matrix. Then, Equation (16) can be optimized to

(21) $$\begin{array}{cc} E(\theta_{f},\theta_{y},\theta_{d}) & =\sum_{\begin{array}{l} i=1,\cdots ,N\\ d_{i}=0 \end{array}}L_{y}(G_{y}(G_{f}(x_{i};\theta_{f});\theta_{y}),y_{i})\\ & +\sum_{i=1,\cdots ,N}L_{d}(G_{d}(R_{\lambda }(G_{f}(x_{i};\theta_{f}));\theta_{d}),y_{i}) \end{array}$$

During the training process, due to the presence of GRL, as the number of training epochs increases, the source domain data and target domain data in the domain classification part become increasingly mixed. Eventually, the model considers the data from both domains as coming from the same domain. This is achieved by setting the source domain as ‘0’ and the target domain as ‘1’, performing a binary classification task. However, the loss function for the classification task provides only a qualitative measure of the differences in features between different domains and does not precisely indicate the extent of distribution differences.

Therefore, this paper introduces the Bhattacharyya distance [26] to optimize the loss function in the domain classification part. The original binary classification task is replaced with a nonlinear approximation of the Bhattacharyya distance using the neural network, allowing for a quantified measure of distribution differences. The advantages of the Bhattacharyya distance function include its typically low complexity, high computational efficiency, probabilistic interpretability, and mitigation of the gradient vanishing problem during training.

For probability distributions P and Q on the same domain X, the expression for the Bhattacharyya distance function is as follows:

(22) $$D_{B}(P,Q)=-\ln(\sum_{x\in X}\sqrt{P(x)Q(x)})$$

where $0\leq \sum_{x\in X}\sqrt{P(x)Q(x)}\leq 1$ and $0\leq D_{B}\leq \infty$. The smaller the Bhattacharyya distance, the more similar the two probability distributions. The improved DANN model based on the above method is the BDANN model.

### 3.3. Construction of Overall Model

The overall model adopts the GNN-LSTM-BDANN network structure (Figure 3), which comprises the feature extraction section $G_{f}$, domain classification section $G_{d}$, and life prediction section $G_{y}$.

The feature extraction section $G_{f}$ is constructed using a combination of two GConv layers, two pooling layers, and LSTM layers, arranged in a sequential stack. It ultimately outputs feature vectors. The domain classification section $G_{d}$ and the remaining life prediction section $G_{y}$ consist of fully connected layers. The domain classification section $G_{f}$ outputs two probability values $\left[p_{s},p_{t}\right]$ ($0\leq p_{s},\;p_{t}\leq 1$ and $p_{s}+p_{t}=1$), representing the likelihood of the data belonging to the source domain dataset or the target domain dataset, respectively. The larger of the two probabilities determines the assigned domain. During backpropagation, the domain classification section undergoes GRL, inverting the error gradients to confuse the source domain datasets and target domain datasets. The loss function employs an approximate fitting of the Bhattacharyya distance, which replaces the loss function of the binary classification task.

The life prediction section $G_{y}$ directly outputs the predicted life value after passing through multiple fully connected layers.

## 4. Experimental Result and Discussion

### 4.1. Data Preparation

This section mainly verifies the performance of the model proposed in this paper. First, we need to acquire sound data through experiments.

As the operational duration of elevator door systems increases, their health gradually deteriorates. Therefore, this study monitors and collects sound data from multiple elevator door systems during operation to obtain degradation process data. One of these elevators is then designated as the target elevator, and historical operation data from other elevators is utilized to predict the RUL of the target elevator door system. The sound data used in this study originates from elevators used in buildings. During the continuous collection of sound data, the sound data of the elevator is collected once a week. When an elevator has been in operation for approximately 160 weeks, it exhibits noticeable difficulties in opening and closing its doors accompanied by noise. Around 180 weeks of operation, the elevator occasionally experiences situations where the doors cannot be opened or closed, indicating a malfunction; at this point, the RUL of the elevator door system is defined as 0. To avoid accidents, the fault should be warned, and maintenance personnel should be arranged for maintenance in advance. In this study, we defined that the elevator door system needs to be repaired when the RUL is 20%. This threshold is also used when utilizing the model for predictions in subsequent analyses.

**Figure 8:**
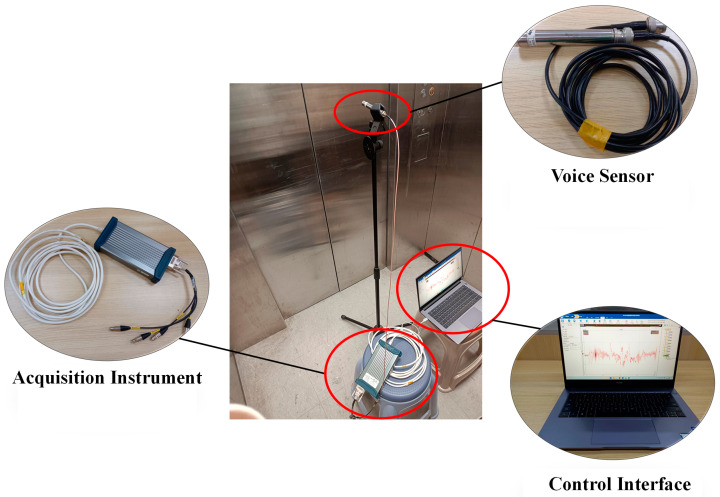
Sound acquisition platform.

**Figure 9:**
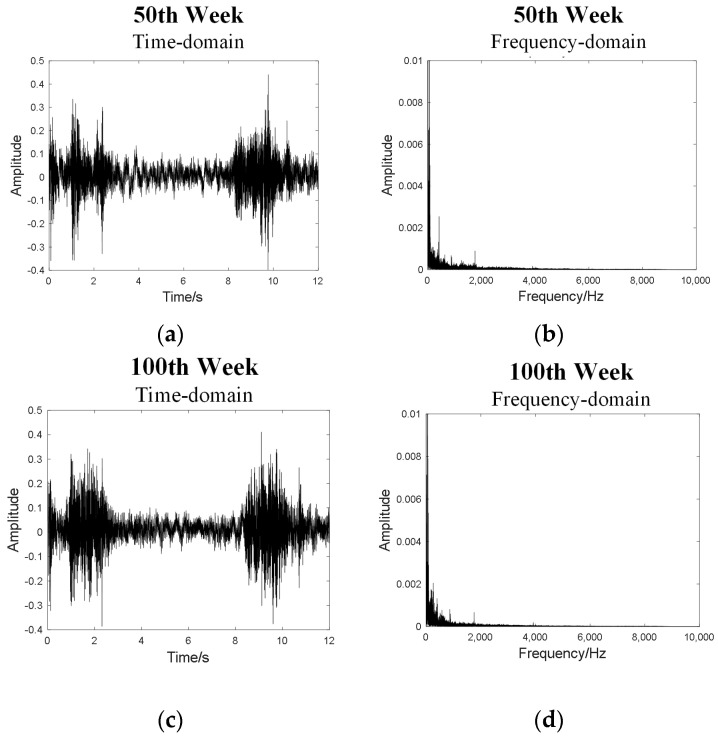
(**a**) Time-domain plot at 50th week; (**b**) Frequency-domain plot at 50th week; (**c**) Time-domain plot at 100th week; (**d**) Frequency-domain plot at 100th week.

The data acquisition platform is shown in Figure 8. The PCB378B02 microphone is used to collect the sound signal when the elevator door opens and closes, place the microphone in the car, connect the acquisition instrument and the computer, the sampling frequency is set to 20 kHz, and each “opening → closing the door” is defined as a set of data; each set of data is 12 s (the time of opening and closing the door), and data is collected once a week. The time-domain and frequency-domain plots of the sound signals from elevator door systems at different operating times are shown in Figure 9, taking the sound data from the 50th and 100th weeks as examples.

**Figure 10:**
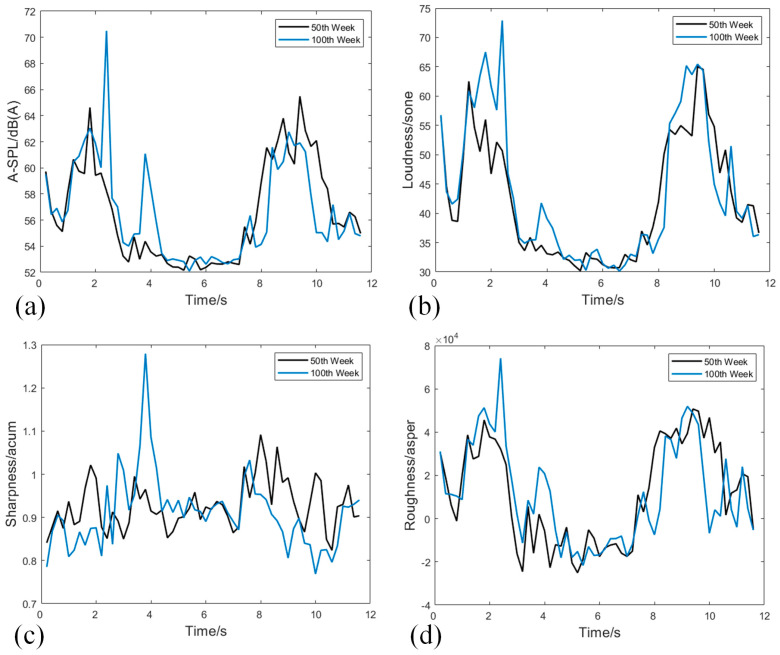
(**a**) Comparison of sound signal A-SPL between 50th week and 100th week; (**b**) Comparison of sound signal loudness between 50th week and 100th week; (**c**) Comparison of sound signal sharpness between 50th week and 100th week; (**d**) Comparison of sound signal roughness between 50th week and 100th week.

It can be observed that it is difficult to directly discern the difference in sound signals from elevator door systems at different operating times in both the time-domain and frequency-domain signals. Therefore, sound quality feature extraction is applied to the sound signals. First, determine the size of the Fourier window, divide the signal by the window function, and then calculate the A-weighted sound pressure level, loudness, sharpness and roughness of the signals in each window. The sound quality features of sound signals from elevator door systems at different operating times are shown in Figure 10. To some extent, these features can reflect the differences between signals.

From Figure 10, it is evident that the feature values of different sound quality features vary significantly in magnitude. Therefore, it is necessary to perform normalization on the entire dataset. Normalization refers to a linear transformation of the original data, bringing the results within the range of 0 to 1, which can enhance the efficiency of the model. The data normalization equation is as follows:

(23) $$X_{norm}=\frac{X-X_{\min}}{X_{\max}-X_{\min}}$$

where $X_{norm}$ represents the normalized data, X represents the original data $X_{\min}$, and $X_{\max}$ represent the maximum and minimum values of the data set, respectively.

After data normalization, it is necessary to further construct the data into graph data that can be learned by GNN. In this paper, the RadiusGraph method is used to construct sound quality feature data set into graph data. In this method, cosine similarity is used to estimate the distance between samples, and the threshold ε is defined. If the cosine similarity is greater than the threshold, there will be an edge between the two nodes. Therefore, the neighbors of node can be represented as

(24) $$Ne(x_{i})=\varepsilon ⊙\;-\;radius(x_{i},\Psi ),\;\;\;if\;\varepsilon ⊙\;-\;radius(x_{i},\Psi )>\varepsilon$$

where ε⊙ − radius(⋅) denotes the calculation of the cosine similarity between node $x_{i}$ and the nodes in the set Ψ and returns the nearest neighbors of node $x_{i}$.

### 4.2. Model Performance Verification

The elevator from which the sound data in the source domain originates is named Elevator S, while the elevator from which the sound data in the target domain originates is named Elevator T. In such a scenario, if training a model using data from one elevator and then applying that model to predict outcomes for the other elevator, the predictions may not be very accurate due to the issue of data dissimilarity. The next step would be to experimentally validate this inference. 

Firstly, the sound quality feature data for the door sound signals of two elevators are calculated using the method described earlier, and graph data is constructed. Elevator S has known life labels and known domain labels, while elevator T has unknown life labels and known domain labels. Subsequently, the graph data is input into the model. After multiple GConv layers and pooling layers to extract abstract features from the dataset, the LSTM is employed to identify temporal variations within these abstract features, resulting in a set of feature vectors. 

For elevator S, the feature vectors are input separately into the life prediction section and the domain classification section. In the life prediction section, the model predicts the life label for this dataset, while in the domain classification section, it predicts which domain this dataset belongs to. For elevator T, only the domain classification section is used to predict which domain this dataset belongs to. When training the model, the goal is to confuse the model between the source domain data and the target domain data. 

Finally, during model testing, the model is tasked with predicting only the life labels of the target domain data for elevator T. This is done to verify whether the model can leverage the knowledge learned from the source domain data to predict the life of the target domain data.

In order to suppress the noisy signal from the domain classifier early in the training process, a dynamically varying adaptation factor $\lambda_{p}$ is used instead of a fixed value:

(25) $$\lambda_{p}=\frac{2}{1+e^{\left(-\gamma *p\right)}}-1$$

where p represents the relative value of the iteration process, which is the ratio of the current iteration times to the total iteration times. Meanwhile, γ denotes the weight parameter, set to 10.

In order to prevent the model from falling into the local optimal solution and avoiding overfitting, the learning rate is also set to transform with the iterative process. The transformation equation is as follows:

(26) $$\mu_{p}=\frac{\mu_{0}}{{\left(1+\alpha *p\right)}^{\beta }}$$

where $\mu_{0}=0.01$ represents the initial learning rate, p denotes the relative value of the iteration process, and α=10 and β=0.75 are hyperparameters.

Next, the excellent performance of the proposed model will be validated from two aspects: (1) the excellence of the feature extraction section; (2) the excellence of the improved transfer learning technique BDANN.

**Figure 11:**
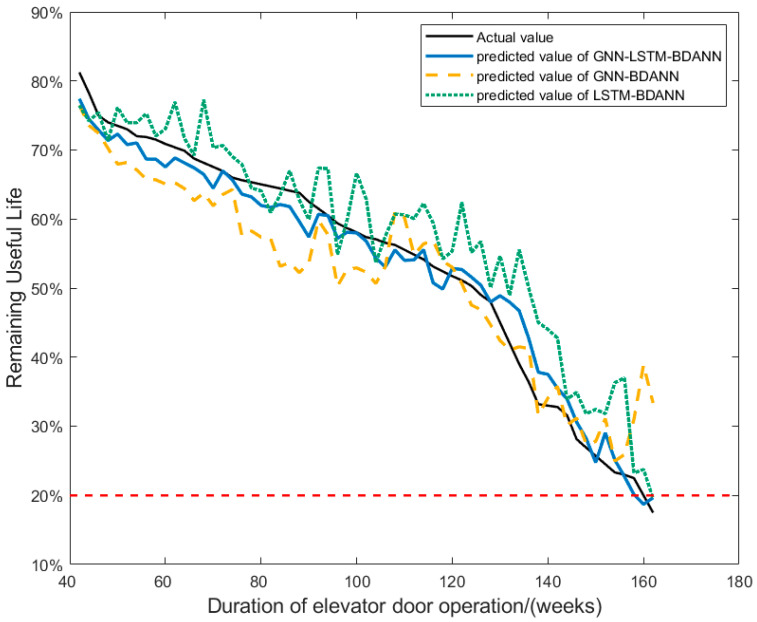
Comparison of model results of different feature extractors.

Firstly, the LSTM-BDANN model using LSTM as the feature extractor, the GNN-BDANN model using GNN as the feature extractor, and the GNN-LSTM-BDANN model are trained. The results of each model are shown in Figure 11. Mean Squared Error (MSE) and Root Mean Squared Error (RMSE) are used as evaluation metrics for the models [27]. Table 1 presents the MSE and RMSE evaluation results for each model.

It can be seen from the results that compared with using LSTM and GNN as feature extractors alone, the model using GNN-LSTM as a feature extractor is more accurate in predicting the results. This is because when extracting potential features from the data, GNN can effectively capture the graph structure relationship between feature data, while LSTM helps to consider the time dependence of feature data. The GNN-LSTM that combines the two can obtain a more representative and rich feature representation, which can improve the sensitivity of the model to the change of system state, so that the model can better capture the state of the predicted object at different time points, and at the same time, it can more comprehensively capture the evolution process of data and predict the degradation trend more accurately. The model results of GNN-LSTM as the feature extractor are more stable than those of the GNN and LSTM independently. The simultaneous capture of graph structure information and time series information in the data helps to reduce fluctuations caused by noise or temporary changes in the data, indicating that the model is more robust to small changes in the input data, which is an important advantage in practical applications. Therefore, it can be concluded that the performance of the GNN-LSTM feature extractor is better than that of GNN and LSTM feature extractors alone.

**Figure 12:**
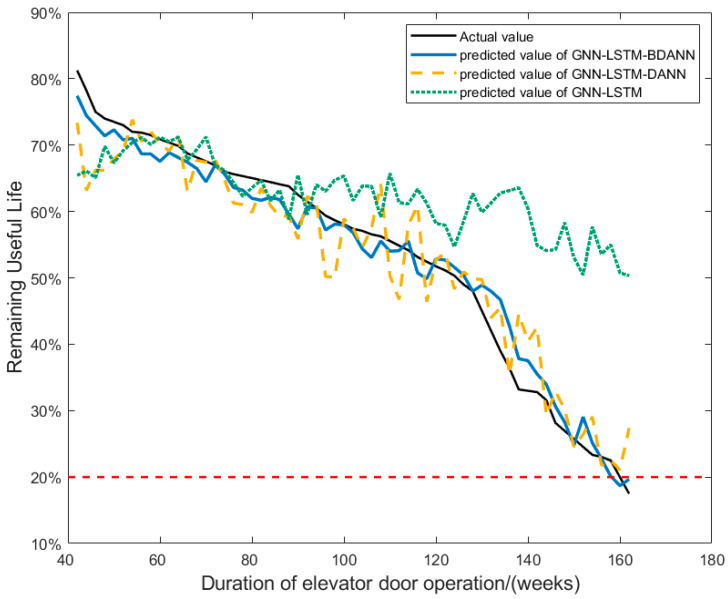
Comparison of the results of the three models.

The GNN-LSTM model without introducing DANN transfer learning, the GNN-LSTM-DANN model without improvements, and the improved GNN-LSTM-BDANN model were respectively used for training. When training the GNN-LSTM model, the elevator S data is used as the training set, and the elevator T data is used as the test set after the training is completed. The results are shown in Figure 12, and Table 2 shows the evaluation metrics of each model.

For the GNN-LSTM model, due to the absence of transfer learning methods, the prediction performance on elevator T after training on elevator S data is not satisfactory. It can even be argued that the model, when trained on elevator S data, has not acquired knowledge applicable to other elevators. For a singular model, predicting RUL across different machines remains a challenging problem that is difficult to overcome.

The results from the GNN-LSTM-DANN model and the GNN-LSTM-BDANN model demonstrate a significant improvement in cross-machine prediction performance with the introduction of transfer learning. Leveraging the specificity of transfer learning enables the model to learn deeper knowledge during training and apply this learned knowledge to other objects. This approach, to a certain extent, addresses the challenge of cross-machine Remaining Useful Life (RUL) prediction for a single model, especially when there are substantial differences in data distribution and fault evolution characteristics.

**Figure 13:**
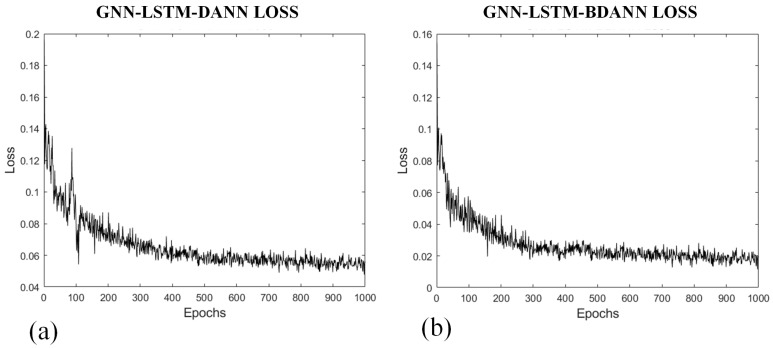
(**a**) Loss function curve of GNN-LSTM-DANN model training; (**b**) Loss function curve of GNN-LSTM-BDANN model training.

In addition, the GNN-LSTM-BDANN model, compared to the GNN-LSTM-DANN model, incorporates improvements in the DANN part of the model. As evident from the descending trends of the loss functions for the two models in Figure 13, the GNN-LSTM-BDANN model has essentially converged by the 300th epoch, while the GNN-LSTM-DANN model converges by the 500th epoch. Therefore, the GNN-LSTM-BDANN model exhibits higher computational efficiency, faster convergence, and lower final loss values, and mitigates the issue of gradient vanishing during training. Its predictive performance evaluation metrics are also slightly superior to the unimproved GNN-LSTM-DANN model.

In summary, it can be concluded that the GNN-LSTM-BDANN model with improved BDANN transfer learning has stronger generalization and higher prediction accuracy than the unimproved GNN-LSTM-DANN model, which can make use of elevator door sound data to predict faults of different elevators and accurately identify their RUL.

**Figure 14:**
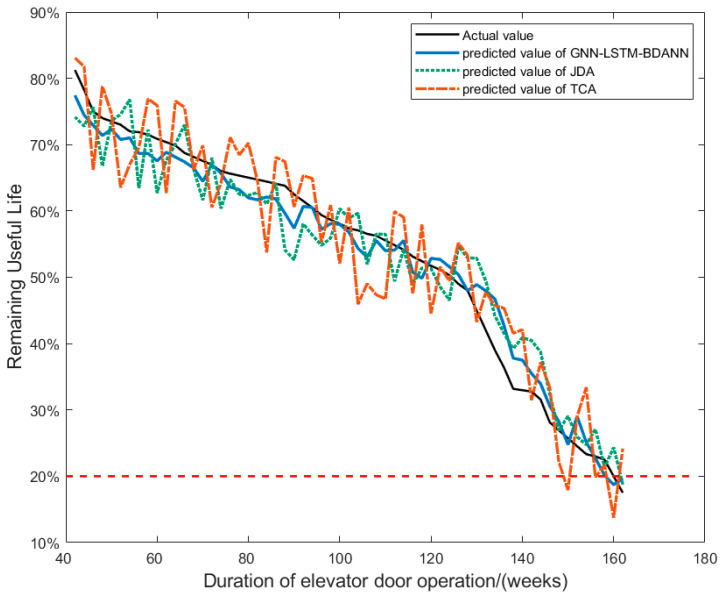
Comparison of results of different transfer learning models.

To further validate the performance of the improved transfer learning model proposed in this paper, comparisons were conducted with two established transfer learning methods: Transfer Component Analysis (TCA) [28] and Joint Distribution Adaptation (JDA) [29]. The core idea behind TCA lies in employing kernel methods or other dimensionality reduction techniques to perform nonlinear transformations on the data from both the source and target domains, mapping them into a new common feature space where efforts are made to render the marginal distributions (i.e., the overall probability distributions across each domain) as close as possible, thereby enhancing the effectiveness of applying knowledge learned from the source domain to the target domain. JDA builds upon this by considering not only the difference in marginal distributions but also the disparity in the joint distributions. Beyond reducing the marginal distribution discrepancies between the source and target domains in the low-dimensional embedded space, JDA introduces adaptations to the conditional distributions (i.e., the feature distributions given class labels), aiming to ensure not only similarity in feature statistical properties across individual dimensions but also the preservation of relationships among different classes across both domains. This approach allows for more effective handling of complex cross-domain distribution shifts. The results are shown in Figure 14, and Table 3 shows the evaluation metrics of each model.

The results indicate that the improved BDANN transfer learning model developed in this research outperforms both the TCA and JDA transfer learning models. The core of DANN lies in its “adversarial training” mechanism, through which the feature extractor in the model can learn some common, domain-independent deep feature representation that is more robust and mobile and can better adapt to the task of the target domain. While TCA primarily seeks to mitigate distribution discrepancies by identifying a shared subspace between the source and target domains, it may fall short of fully addressing deeper, complex distribution shifts.

JDA, although advancing on TCA by considering joint distribution adaptation conditioned on class labels, might be limited in practical applications due to stringent assumptions and potentially inadequate adaptability to high-dimensional, nonlinear feature distributions. In the context of this study’s problem of predicting the remaining useful life of elevator doors, the improved BDANN is more effective at uncovering informative patterns in the sound signals collected during elevator door operations, focusing on the intrinsic rules that critically influence the RUL predictions.

In summary, the GNN-LSTM-BDANN model constructed in this work can be regarded as superior to TCA and JDA transfer learning methods when applied to the task of transfer learning on elevator door sound data. Its unique adversarial training approach, coupled with its potent domain adaptation capabilities, leads to increased accuracy in predicting the RUL of elevator doors.

## 5. Conclusions

In this paper, a novel fault prediction approach termed GNN-LSTM-BDANN is proposed, which integrates the transfer learning to predict the RUL of door systems of the elevators with a focus on failures caused by wear in guide rails, rollers, bottom tracks, and slides. The methodology initially merges GNN with LSTM as the feature extraction module of the model. It employs GNN to extract high-level features from the sound quality data, followed by LSTM to discern the temporal patterns within these features, thus generating a set of representative feature vectors. These vectors are subsequently fed into both the life prediction component and the domain classification section of the model. By deliberately blurring the distinction between source and target domain data, the method facilitates knowledge transfer in deep learning architectures. Experimental validation is conducted using an array of elevator door sound signal datasets, which are input into the proposed model for training and testing, thereby assessing its performance. The main conclusions are as follows:

(1) Contrasted with conventional deep learning techniques, the Graph Neural Network (GNN) is capable of revealing intricate relationships between data points. When integrated with Long Short-Term Memory (LSTM), it effectively captures more profound time-series characteristics. The GNN-LSTM feature extraction module proposed in this study outperforms standalone GNN or LSTM methods in terms of feature extraction efficacy.
(2) The BDANN transfer learning method proposed in this paper, as compared to the original DANN, addresses the issue of DANN’s inability to precisely represent distribution differences in different domains. It alleviates the problem of gradient vanishing during training, thereby enhancing model stability.
(3) The experimental results substantiate that the GNN-LSTM-BDANN prediction model proposed in this study effectively harnesses historical data from other elevators to realize the prediction of the remaining service life for the target elevator, and it exhibits superior predictive performance compared to the two transfer learning methods, TCA and JDA. Consequently, this model provides a favorable foundation for the prediction of failures and the implementation of preventive maintenance in elevator door systems.

## Figures and Tables

**Table 1:** Three models evaluation metrics of different feature extractors.

	MSE	RMSE
GNN-LSTM-BDANN	0.0036	0.0601
GNN-BDANN	0.0237	0.1540
LSTM-BDANN	0.0318	0.1783

**Table 2:** Three models evaluation metrics.

	MSE	RMSE
GNN-LSTM-BDANN	0.0036	0.0601
GNN-LSTM-DANN	0.0044	0.0666
GNN-LSTM	0.0626	0.2502

**Table 3:** Different transfer learning models evaluation metrics.

	MSE	RMSE
GNN-LSTM-BDANN	0.0036	0.0601
TCA	0.0183	0.0855
JDA	0.0170	0.0825

## Data Availability

Data are contained within the article.
